# Supplementary material for: Embodied time: Effect of reading expertise on the spatial representation of past and future
Source: PLoS One. 2022 Oct 27;17(10):e0276273. doi: 10.1371/journal.pone.0276273 (PMC9612582; doi:10.1371/journal.pone.0276273)
Supplement: Attachment — Submitted filename: Response to Reviewers.docx [file pone.0276273.s001.docx]

Response to editorial revision requests (in blue)

Dear Editor,

We are very grateful to the editor and reviewers for their suggestions and comments which substantially helped Improve our manuscript entitled “Embodied time: Effect of reading expertise on the spatial representation of past and future”. Please find below a summary of how we addressed each point.

Reviewers' comments:

**Reviewer #1:**

1. Although I have nothing against the embodied interpretation of the reported effects, I think there is at least one main alternative interpretation that should be explicitly mentioned. Such interpretation has more simply to do with stimulus-stimulus and stimulus-response compatibility (for similar phenomena on up/down, small/big semantic properties, see, e.g., Santens & Gevers (2008), Cognition; Sellaro et al. (2015), Psychological Research; Treccani et al. (2019), Frontiers in Psychology). These phenomena would originate from task-related factors and may be read in the framework of polarity (e.g., Proctor & Xiong (2015), Current Direction in psychological Science). To my view, this interpretation is simpler, with one basic mechanism able to account for the pattern of data from many experiments. Although the authors may obviously keep the theoretical framework they prefer, I think they should introduce this alternative proposal at least to interpret their findings and also try to explain why their interpretation would be preferable. Similarly, some of the empirical findings investigating compatibility effects and not referring to the embodied perspective to frame their study should be (at least) cited.

RESPONSE: We thank the reviewer for this suggestion. We added the references to the polarity account and a discussion of our effects in terms of polarity. It should be noted, however, that Santiago and Lakens (2015) explicitly tested the predictions of the polarity hypothesis in a series of four experiments. Their Experiment 4 was a past-future categorization task, just like ours, and their results did not support the predictions in terms of polarity (see also Santiago, 2007). Nevertheless, as suggested, we added a discussion of our results in terms of the polarity account (see p.471-501).

2. Throughout the manuscript the authors use different labels to refer to (likely) the same thing. In particular, when referring to motor action, they use different terms, as, e.g., motor execution and motor planning. However, different terms may refer to different things – to keep the example, motor planning is a higher-order level of processing that starts earlier than motor execution and in which the to-be-executed motor commands are selected and set up. I think the authors should be consistent in the terminology adopted and precisely clarify to what they refer to.

RESPONSE: We thank the reviewer for spotting this inconsistency. We have clarified the use of our terminology and now use motor action consistently throughout the manuscript.

3. Throughout the manuscript the authors (overtly and/or covertly) seem to propose the equation motor response (i.e., manual responses) = motor network. However, I find this equation too speculative: a) what the authors call motor congruency seems to be a spatial congruency effect, exactly as the one represented by stimuli dislocation on the screen (on this issue, see also my point above). The motor system is in no way (directly or indirectly) involved in the experimental manipulation. It is simply another spatial manipulation, with responses given either on the left or right side of the keyboard. Following on this line, the authors repeatedly state that their findings support that abstract temporal concepts are represented in both spatial and motor networks. Maybe I’m missing something, but I struggle to understand on what data these conclusions are based on. The authors report some spatial compatibility effects, with no measure or manipulation directly targeting the activity within the spatial and/or motor network. The data, instead, seem to me easily accountable for in the framework of the polarity principle (e.g., Proctor & Cho (2006), Psychological Bulletin; Proctor & Xiong (2015), Current Direction in psychological Science). Note that I’m not saying that the authors must embrace this interpretation, but simply that they should not over-interpret their data.

RESPONSE:

We actually addressed this very point in our previous study, published In JEP:LMC (Grasso et al. 2021), where we contrasted spatial compatibility effects without movement (button presses) and spatial compatibility effects with movement. The MTL effects were present only when the decision involved movement, which is why we referred to motor networks in reference to our previous work. Note also that the contrast between static responses (without movement, button presses) and dynamic responses (with movement) is actually a test of the polarity hypothesis.

However, we agree that the terms used in the current manuscript are potentially ambiguous. We have now replaced motor network by motor system. In addition, we would like to clarify that we manipulated two types of space-time congruency in our experiment: i) the congruency between the temporal content of the stimuli and their spatial location on the screen, which we labelled 'spatial congruency' because this manipulation is not dependant on the side of keyboard responses, and ii) the space-time congruency of the response hands on the keyboard, independently of the spatial location of the stimuli on the screen, which we labelled 'keyboard congruency’. Note also that the congruency effect was stronger when the space-time congruency condition was induced by motor responses rather than by the location of the stimuli on the screen. This finding was taken to suggest that the localisation of the motor response (and not the spatial localisation of the stimuli) induced the congruency effect. We now explain this point more clearly in the manuscript.

4. Task
   The adopted task is quite explicit. Why didn’t the author use a more indirect task (as, e.g., lexical decision), or at least one less focused on the temporal dimension (e.g., a semantic categorization on an irrelevant dimension)? Can the authors exclude any specific contribution of the task to the reported effect?

RESPONSE:

In our previous work published in JEP:LMC (Grasso et al., 2021), we did in fact use an implicit task (i.e., lexical decision) and we showed that dynamic motion played a key role in the emergence of the MTL effects. By contrast, the main goal of the present study was to determine the relative contribution of motor (keyboard compatibiility) and visual (screen compatibility) processing on the emergence of MTL congruency effects in an explicit temporal task (see introduction).

5. - Experimental Design

I struggled to figure out the exact experimental design, and at the end I’m not sure I came out with the correct one. If I correctly understand, the authors have 3 factors, which are: a) Spatial congruency (i.e., the congruency between the semantic of the verb (past/future) and the position of the stimulus on the screen (left/right)); b) action congruency (i.e., the congruency between the semantic of the verb (past/future) and the side of the keyboard to release the response (left/right); c) Simon congruency (i.e., the congruency between the side of the response and the position of the stimulus on the screen (e.g., a verb at the future presented on the left side of the screen when the response is given pressing a button with the left hand on the left side of the keyboard)).

I have tried to understand all the possible combinations of the factors to understand what items belong to each cell of the design, but I couldn’t figure out the content of some cells. In particular, considering the Simon-congruent condition, what trials would be in the cell Spatial incongruent AND Action congruent, and in the cell Spatial congruent AND action incongruent? Similarly, for the Simon-incongruent condition, what trials would be in the cell Spatial congruent AND Action congruent, and in the cell Spatial incongruent AND action incongruent?
Is the design well balanced? Please clarify, also perhaps by adding a table with an example for each cell of the design.

Further, it is not clear to me why the authors did not try to disentangle the different compatibility effects by manipulating one at the time (and perhaps running two experiments, one per manipulation, with the same participants).

RESPONSE: We agree with the reviewer that the experimental design is complex, and we have tried to make it clearer in the manuscript. The Simon congruency was not manipulated orthogonally but instead covaried with the other two congruency manipulations. Thus, we controlled for Simon congruency, but we did not manipulate it. All the experimental conditions are presented in the figure above. Note that we show conditions for the left side of space only. Conditions are strictly identical for the right side but are not shown for reasons of parsimony. We have added a sentence to explain this in the description of the figure.

To clarify, when keyboard responses are congruent (condition A and B), spatial congruency and Simon congruency are always confounded (congruent Simon = congruent spatial; incongruent Simon = incongruent spatial). Conversely, when keyboard responses are incongruent: Simon and spatial congruency oppose one another: if Simon is congruent, spatial screen location is incongruent and vice versa.

- Simon-congruent, Spatial incongruent AND Action congruent: this is not a possible combination.
- Simon-congruent, Spatial congruent AND action incongruent: condition D.
- Simon-incongruent, Spatial congruent AND Action congruent: this is not a possible combination.
- Simon-incongruent, Spatial incongruent AND action incongruent: this is not a possible combination.

Further, we voluntarily choose to manipulate keyboard congruency in two different sessions (within-participants) at an interval of two weeks For example, in the first session, a given participant used an MTL-congruent response mapping while in the second session the participant used an MTL-incongruent response mapping.

We tried to make all this point clearer in the method section.

6. Minor points
   - What is the distribution of reading expertise? Are there also participants with relatively low level of expertise or the variable has a skewed distribution toward the relatively high expertise?

RESPONSE: The SLS score is based on the number of sentences correctly read/judged in 3 minutes (max 77 sentences). We obtained a very good distribution of reading abilities ranging from 11.0 to 67.00 correctly judged sentences, with an average of 28.07. The distribution was somewhat skewed, with most participants having a medium level of expertise but a few having very high levels of expertise. However, a non-normal distribution of one of the predictor variables in a mixed-effect model is not a problem as long as the residuals of the fitted variable are normal, which is the case. Please find the distribution of SLS scores (figure1) and plots of the model assumptions in figure 2. This is now acknowledged.

Figure 1. Distribution of reading expertise (expressed by the SLS standardised score). SLS scores were standardized using the scale() function in R, which centers and scales the columns of a numerical matrix. Higher values reflect greater reading expertise.

Figure 2. Visual check of the various model assumptions (normality of residuals, normality of random effects, linear relationship, homogeneity of variance, multicollinearity), fitted with the check_model() function of the package performance (Lüdecke et al., 2021).

- p. 14, ls. 357-358, check the statistics reported in parentheses.

RESPONSE: we corrected the corresponding statistics.

- At the osf link, I couldn’t find the script of the analyses.

RESPONSE: we thanks the reviewer for pointing out this mistake and have now added the scripts. of the statistical analyses.

**Reviewer #2:**

Two general considerations:

1. It would be useful to say from the start that the study is a replication one;
2. b) it would be helpful to clarify from the start (and in the abstract) that the task consists of a temporal categorization one.

RESPONSE: done

- Page 3. When discussing abstract concepts, the authors might want to refer to authors who classified them as challenging for embodied cognition views, e.g., Borghi et al., 2017, The challenge of abstract concepts; G. Dove, 2021, The challenges of abstract concepts.

RESPONSE: done.

- Page 3, line 75., The authors might want to refer more extensively to the seminal work of Lera Boroditsky, quite important in this area.

RESPONSE: done.

- Line 97-on. When describing the procedure of the experiment by Grasso et al., please clarify that the words refer to time.

RESPONSE: done.

- Line 129 (minor). Please substitute Casasanto et al. with Casasanto and colleagues.

RESPONSE: done.

- Line 196. I think the authors should tell us more about how they selected the sample and determined the sample size.

RESPONSE: We added more information about the selection of the sample. As concerns the sample size, we initially aimed for 100 participants (final inclusion of 91), which is largely sufficient to detect an effect size of d=.3 (found in our previous experiment with an implicit task) with a power of over 80% (see Brysbaert, 2019)

- Sample. In the sample, gender is unbalanced: might this have affected the results? How about education level? The authors could add some considerations on how demographic elements might have influenced the results.

RESPONSE: Almost half of the participants were women (83 participants, 36 women) and, to our knowledge, no available data seems to suggest a gender effect. Concerning the laterality of the participants, this variable was tested and had no effect. We had no information on the education level of the participants.

Line 205. Technically, the Helsinki declaration would require the work to be preregistered, and this experiment is not.

RESPONSE: We thank the reviewer for pointing this, we removed this sentence.

- Conclusion – In the conclusion, I think the authors should return to what was introduced at the beginning of the paper, i.e., the implications of their work for studies on abstract concepts.

RESPONSE: We thank the reviewer for this suggestion. We agree that this part was missing, so we have added a few sentences in discussion (see l. 587-591).

REFERENCES

Brysbaert, M. 2019 How Many Participants Do We Have to Include in Properly Powered Experiments? A Tutorial of Power Analysis with Reference Tables. Journal of Cognition, 2(1): 16, pp. 1–38. DOI: https://doi.org/10.5334/joc.7

Lüdecke et al., (2021). performance: An R Package for Assessment, Comparison and Testing of Statistical Models. Journal of Open Source Software, 6(60), 3139. https://doi.org/10.21105/joss.03139
