# Supplementary material for: Embodied time: Effect of reading expertise on the spatial representation of past and future
Source: PLoS One. 2022 Oct 27;17(10):e0276273. doi: 10.1371/journal.pone.0276273 (PMC9612582; doi:10.1371/journal.pone.0276273)
Supplement: Attachment — Submitted filename: Response letter - Plos one 2709.docx [file pone.0276273.s002.docx]

Response to revision requests (in blue).

Dear Dr. Barca,

Many thanks for giving us the opportunity to provide a second revision of our manuscript. Please find enclosed the revised version of our manuscript entitled “Embodied time: Effect of reading expertise on the spatial representation of past and future”. We are very grateful to the editor and reviewers for their truly positive suggestions and comments. Please find below our response to each point.

Journal Requirements:

Please review your reference list to ensure that it is complete and correct. If you have cited papers that have been retracted, please include the rationale for doing so in the manuscript text, or remove these references and replace them with relevant current references. Any changes to the reference list should be mentioned in the rebuttal letter that accompanies your revised manuscript. If you need to cite a retracted article, indicate the article’s retracted status in the References list and also include a citation and full reference for the retraction notice.

Reviewers' comments:

Reviewer's Responses to Questions

**Comments to the Author**

1. If the authors have adequately addressed your comments raised in a previous round of review and you feel that this manuscript is now acceptable for publication, you may indicate that here to bypass the “Comments to the Author” section, enter your conflict of interest statement in the “Confidential to Editor” section, and submit your "Accept" recommendation.

Reviewer #1: (No Response)

Reviewer #2: All comments have been addressed

2. Is the manuscript technically sound, and do the data support the conclusions?

The manuscript must describe a technically sound piece of scientific research with data that supports the conclusions. Experiments must have been conducted rigorously, with appropriate controls, replication, and sample sizes. The conclusions must be drawn appropriately based on the data presented.

Reviewer #1: Partly

Reviewer #2: Yes

3. Has the statistical analysis been performed appropriately and rigorously?

Reviewer #1: Yes

Reviewer #2: Yes

4. Have the authors made all data underlying the findings in their manuscript fully available?

The [PLOS Data policy](http://www.plosone.org/static/policies.action#sharing) requires authors to make all data underlying the findings described in their manuscript fully available without restriction, with rare exception (please refer to the Data Availability Statement in the manuscript PDF file). The data should be provided as part of the manuscript or its supporting information, or deposited to a public repository. For example, in addition to summary statistics, the data points behind means, medians and variance measures should be available. If there are restrictions on publicly sharing data—e.g. participant privacy or use of data from a third party—those must be specified.

Reviewer #1: Yes

Reviewer #2: Yes

5. Is the manuscript presented in an intelligible fashion and written in standard English?

PLOS ONE does not copyedit accepted manuscripts, so the language in submitted articles must be clear, correct, and unambiguous. Any typographical or grammatical errors should be corrected at revision, so please note any specific errors here.

Reviewer #1: Yes

Reviewer #2: Yes

6. Review Comments to the Author

Please use the space provided to explain your answers to the questions above. You may also include additional comments for the author, including concerns about dual publication, research ethics, or publication ethics. (Please upload your review as an attachment if it exceeds 20,000 characters)

Reviewer #1: I'm one of the reviewers of the previous round. I think the authors have done a good job in addressing most of my criticisms. I still have two concerns.

The first one is related to the experimental design. The authors have now better clarified their design. If I'm correct, in half cases (i.e., when keyboard responses are congruent) Simon condition is confused with the spatial condition, whereas in the other half cases (i.e., when keyboard responses are incongruent), Simon and spatial conditions are distinguishable. If this is the case, don't the authors think that this is problematic in terms of interpreting their findings? (In half trials, two types of congruency/incongruency are jointly at work, whereas in the other half, only one at time). Take, for example, figure 3: It shows the Simon congruency effect for the keyboard congruent and the keyboard incongruent condition.

RESPONSE: Yes, this is a fundamental limitation of the original Santiago et al. design used in the present study. In fact, we had addressed this confound in our previous research (Grasso et al, 2021) by presenting words in the center of the screen and asking participants to make left or right spatially directed movements. However, because our aim in the current study was to investigate both spatial congruency on the screen and spatial congruency of the keyboard (independently of one another) lateralization of both stimulus presentation and response hand had to be orthogonalised, which inevitably leads to a confound with the Simon effect. Unfortunately, there is no way to manipulate these three effects orthogonally. Thus, the best one can do is to control for the Simon effect by adding it to the statistical mixed effect model ( Model <- lmer(Data$ReverseRT ~ SLS_CORRECTE_s * Simon_Congruency + Keyboard_Rep * SLS_CORRECTE_s + Screen_Congruency * SLS_CORRECTE_s + Age + (1|New_ID)+(1|Item)) ) and being cautious in the interpretation of results. Including the Simon effect in the statistical model effectively partials out the part of the variance that is due to this variable. Also note that the analysis showed no interaction between screen congruency and Simon congruency and no interaction between keyboard congruency and Simon congruency (models were tested and compared using the anova() function of the lmerTest package for model comparison) . Thus, we are fairly confident that our results are not due to a confound with Simon. As requested, we mention this as a limitation of the design, and we interpret our results with caution.

However, for the keyboard congruent condition, is it the Simon or the spatial condition (or both)? Although there is no main effect of space-time congruency of screen location, can we be sure that such variable does not exert any role when co-occur with the Simon condition?

RESPONSE: As we can see in the statistical analyses, there was no significant interaction between each type of congruency. Given that keyboard congruency did not interact with either screen or Simon congruency, any effect of screen or Simon congruency in just one of the keyboard conditions cannot be meaningfully interpreted. As a reminder, the keyboard congruency effect (congruent vs. incongruent) was completely orthogonal to Screen congruency and Simon congruency. Thus, regardless of Screen or Simon congruency/incongruency, RTs were slower in the incongruent keyboard condition (grouping conditions D and C) than in the congruent keyboard condition (grouping conditions A and B). This can be seen in the graph below.

The second one is related to some of my previous comments. In particular, I raised doubts about the need to bring up the motor system to explain the reported findings. The authors replied that an “abstract compatibility” interpretation does not hold and they already published a study proving the involvement of the motor system. The authors have (almost) convinced me on the goodness of their interpretation (although I still find it not very economic), but I would see a bit more caution on the way they embrace it. I still don't see strong proof in favor of the involvement of the motor system. The authors refer to their previous study (Grasso et al., 2021), which I decided to read to have a more complete view of their work. Although that study has an experiment directly manipulating the involvement of the motor system (Experiment 2), I have found the results of such experiment not fully convincing. In fact, in the ANOVA analysis, they report a strong three-way interaction between time and side (i.e., the congruency effect), but a weak three-way interaction between time, side and device (which should be the critical result, but it is significant by participants, but not by items). This suggests that the device does not play a so strong role in modulating the effect. The three-way interaction becomes fully significant only when the two conditions requiring movements (trackpad and mouse) are collapsed and jointly compared against the keyboard condition (which does not require movement). However, it seems to me that there are no post-hoc comparisons, which makes hard to fully appreciate the pattern. This is also what happens in the mixed-effects model analysis. Also, looking at Figure 2, it seems that keyboard and mouse behave similarly, with the former showing a smaller (but not negligible) effect. To make a long story short, it seems to me that, although the motor component seems to play a role, it does not seem to be necessary to make the effect arise. I would suggest to slightly weaken some of the theoretical statements on the role of motor component.

RESPONSE: We agree with the reviewer that there must be more to the effect than the involvement of spatially directed movements (motor system). Otherwise, no effect would be observed in explicit timing tasks with static keypress responses (see for example the meta-analyses von Sobbe et al. 2019). It is extremely difficult to obtain significant triple interactions by subjects and items and you correctly point out that the triple interaction in the ANOVA analyses was only significant by subjects and items when we collapsed the two movement conditions together (Note, however, that degrees of freedom for items were quite low, 79). Nevertheless, when subjects and items were used as random variables in the equivalent mixed effect model (which has become the standard for analyzing psycholinguistic experiments), the triple interaction was significant. Nevertheless, we decided to follow your request and slightly weakened some of theoretical statements on the role of motor components (see l.45-46, l.427, l.442, l.466, l.486, and l.491).
